# Rating the Acting Moment: Exploring Characteristics for Realistic Portrayals of Characters

**DOI:** 10.3389/fpsyg.2020.615311

**Published:** 2021-02-04

**Authors:** Maria Eugenia Panero, Ellen Winner

**Affiliations:** ^1^Yale University, New Haven, CT, United States; ^2^Department of Psychology and Neuroscience, Boston College, Chestnut Hill, MA, United States; ^3^Harvard Project Zero, Harvard University, Cambridge, MA, United States

**Keywords:** dissociation, flow, empathy, theater, actors, acting, theatre

## Abstract

Good actors appear to become their characters, making them come alive, as if they were real. Is this because they have succeeded in merging themselves with their character? Are there any positive or negative psychological effects of this experience? We examined the role of three characteristics that may make this kind of merging possible: dissociation, flow, and empathy. We also examined the relation of these characteristics to acting quality. Acting students (*n* = 44) and non-acting students (*n* = 43) completed a dissociation measure, and then performed a monologue that was recorded and rated on the dimensions of acting. Participants were then reassessed on dissociation to determine whether it increased as a function of performance. They were also then assessed on flow and empathy. Actors did not differ from non-actors on dissociation, but did score significantly higher than non-actors on some flow and empathy subscales, indicating a positive psychological experience and outcome. While non-actors’ dissociation marginally increased post-performance, actors’ dissociation rose significantly, which could indicate a negative psychological experience. Surprisingly, acting ratings were unrelated to the levels of dissociation, flow, or empathy. We concluded that, while these are tools used by actors to immerse themselves fully in their characters, they may not be necessary to create the illusion of an imaginary character come to life on stage.

## Introduction

Audiences are eager to consume acting – as evidenced by the millions of dollars grossed by major motion pictures and Broadway productions. Despite our fascination with acting, psychologists have performed far fewer empirical studies on this phenomenon than on other art forms (see Winner, 2006, for a review). In the thousands of years since the ancient Greeks are said to have invented theater (Wilson and Goldfarb, 2018), myriad approaches to acting have emerged. Western theater practitioners have long debated whether actors should feel the same emotions as their characters in order to portray them realistically and whether this experience positively or negatively affects the actor.

Based on the teachings of Konstantin Stanislavski, Lee Strasberg and his colleagues developed Method acting in the early 1900s (see Panero, 2019, for a detailed history). This approach to acting is believed to have dominated acting schools ever since, especially in the United States (Konijn, 1997; Krasner, 2000; Knowles, 2004; Lutterbie, 2011; Kemp, 2012; Ohikuare, 2014). Sometimes referred to as “inside out” training, Method acting calls for the actor to feel what the character is feeling.

The actor’s task is to create that level of belief on stage, so that the actor is capable of experiencing the imaginary events and objects of the play with full complement of those automatic physiological responses which accompany a real experience (Strasberg, 1987, p.132).

Many believe that Method acting is psychologically dangerous because acting teachers push their students to feel the emotions of their characters without knowing the risks involved (Konijn, 1997; McFarren, 2003). Some Method actors even strive to stay in character at all times, including in between rehearsals and performances (Konijn, 1997; Benedetti, 1999; Krasner, 2000; Gordon, 2006). Heath Ledger, for example, allegedly inhabited his character the Joker for the movie *The Dark Knight* for 2 months before filming began. He reportedly locked himself in his apartment or hotel room for up to 6 weeks, sleeping only 2 hours per night, and walking around “like a madman” to immerse himself in the character at a “whole new level” (Lyall, 2007; Freyer, 2012; Buitenhuis and Murray, 2017; Timoney, 2017). After Ledger’s death, many speculated that his dedication to Method acting contributed to his overdose, although his family denies this theory (White, 2017).

No systematic studies have yet determined the most popular approach to acting. However, it would be difficult to evaluate the prevalence of “pure” Method acting because many acting training programs have integrated Method acting with Method-like and other acting techniques (Konijn, 1997). Nevertheless, the primary goal of acting training in the United States remains to entangle the actor with the inner life of their character (Chambers, 1997; Krasner, 2000; McFarren, 2003).

The cognitive processes involved in realistically portraying a character are not well understood. In this exploratory study, we present the possibility that dissociation (a potentially negative experience), flow (a positive experience), and empathy (an important social skill) are involved in acting. We examined whether acting students who endorse a Method-like approach score higher than non-acting students on these characteristics, and their potential relationship to the quality of acting. These three constructs were chosen because of their face-value similarity to certain aspects of acting, as described in the following sections. Each of them have been separately examined retrospectively in actors (Martin and Cutler, 2002; Nettle, 2006; Goldstein et al., 2009–2010; Thomson et al., 2009; Panero, 2015), dissociation and flow have been simultaneously examined retrospectively in acting students (Panero et al., 2020) and in dancers (Thomson and Jaque, 2012a). This is the first study to examine the relationship of all three of these characteristics in the same sample of actors immediately following a performance.

### Why Dissociation?

When actors “become” a character, they may be to some extent intentionally dissociating from themselves. *The Diagnostic and Statistical Manual of Mental Disorders* (American Psychiatric Association, 2013) defines dissociation as a break or interruption in the ordinary integration of behavior, body representation, consciousness, emotion, identity, memory, motor control, or perception. Dissociative disorders include dissociative identity disorder (presence of more than one personality), dissociative amnesia (inability to recall important autobiographical information), depersonalization (feeling unreal or detached from the self), and derealization (feeling of unreality or detachment from one’s surroundings). Dissociative experiences may range from these major forms of psychopathology to normative dissociation (e.g., absorption, day or night dreaming, and fantasizing; Butler, 2006; Perez-Fabello and Campos, 2011).

Actors practice re-living personal experiences similar to those of their characters’ in order to feel real emotions in an imagined (or “unreal”) situation. This exercise helps actors to “become” their characters and resembles a deliberate depersonalization and derealization. Professional actors (including those with specific training in Stanislavski’s teachings) score higher than non-actors on dissociation (Thomson et al., 2009; Thomson and Jaque, 2011, 2012b). Student actors score significantly higher on dissociation than the normal population and significantly higher than the cut-off score for dissociative disorders (Panero, 2015; Panero et al., 2020). These findings suggest either that acting training teaches its students to dissociate or that individuals with the capacity to dissociate self-select into acting training.

The studies mentioned above measured dissociation retroactively. Therefore, participants may have inaccurately remembered their experiences or allowed their assumptions about acting to affect their responses. To minimize misremembering and response demand, the current study measured participants’ baseline and post-performance dissociation.

### Why Flow?

When actors say they are “in the moment” or “in character” (i.e., fully embodying the imaginary circumstances and characters of the script), they are likely in a state of flow – the term coined by Csikszentmihalyi (1975) referring to peak positive psychological experiences. One can experience flow during any kind of activity where one’s level of skill meets the level of challenge – from listening to music, playing chess, or running, to intense thinking that leads to scientific discoveries (Csikszentmihalyi, 1975, 1996). When skill outweighs the challenge, boredom occurs; when the challenge outweighs skill, anxiety occurs. Flow is an intrinsically rewarding experience, which results from reaching high levels of concentration and a loss of self-consciousness, and leads to feeling at one with the chosen activity (Csikszentmihalyi and Csikszentmihalyi, 1988; Logan, 1988; Csikszentmihalyi, 1990; Jackson and Kimiecik, 2008).

Flow has been theorized to play a major role in performance art (Jackson and Eklund, 2004), and Martin and Cutler (2002) reported that flow experiences in acting students occurred an average of four times per year. Using qualitative interviews, Allen (2001) found that professional actors described their acting experiences in terms similar to the dimensions of flow: heightened clarity about their needs and intentions, suspension of critical judgment, release from constraints of time, alignment of self with intentions, heightened energy, and satisfaction. Panero et al. (2020) showed that the flow dimension of transformation of time (experiencing time as slowing or speeding up) predicted dissociation scores in acting students. Additionally, the flow dimension of unambiguous feedback (knowing that the current activity is on track toward a desired goal) predicted the absorption and imaginative involvement components of dissociation in acting students. Although these shared dimensions do not mean that dissociation and flow are the same constructs, they provide some evidence of a relationship between flow and dissociation, and that acting may induce both dissociative and flow experiences.

As with dissociation, the studies above measured retrospective flow experiences while acting, and this could lead to erroneous or biased responding. The present study measured participants’ flow immediately following a performance.

### Why Empathy?

Empathy is a vehicle by which actors can see their characters’ life circumstances as part of their own real life. This strategy aims to remove evaluative judgments and integrate a character’s words and behaviors into the actor’s own performance (Gallagher and Gallagher, 2019). Researchers theorize that acting fosters empathy because of the frequency with which actors embody different characters and take on their points of view (Musiker, 2015; Gross, 2018). Since actors frequently portray the personalities of various characters with each new role that they play, it is plausible that highly empathic individuals are drawn to acting. Actor Claire Danes once described her career as that of a “professional empath” (Galanes, 2015).

When discussing empathy, it is important to distinguish between emotional, cognitive, and compassionate empathy. Emotional empathy refers to *feeling* what someone else is feeling. Cognitive empathy refers to *knowing* what someone else is feeling, and is similar to perspective taking (Johnson, 2012). Compassionate empathy refers to helping alleviate someone’s suffering, and is similar to sympathy (Coplan, 2004).

Correlational studies show that professional actors (Nettle, 2006) and student actors (Goldstein et al., 2009–2010) have higher levels of empathy than do non-actors. Goldstein et al. (2009–2010) found that acting students performed significantly better than non-acting students in cognitive, but not emotional or compassionate empathy.

Dumas et al. (2020) measured compassion as a subscale of the Agreeableness dimension of the Big 5 (DeYoung et al., 2007) and showed that actors scored higher than non-actors. When compared across types of actors, student actors reported more compassion than professional actors. The authors argued that, along with the Openness to Experience dimension, compassion allows actors (both student and professional) to develop and embody the characters that they perform.

Two studies examined whether a dose of acting training improves empathy. Chandler (1973) recruited boys diagnosed as anti-social or delinquent and who scored low on a measure of perspective-taking ability (i.e., cognitive empathy). After 10 weeks, the boys in the acting condition (in which they acted in a skit many times) improved more in their cognitive empathy than did the boys in the non-acting conditions.

Goldstein and Winner (2012) studied two groups of children and adolescents. For each age group, participants self-selected into either an acting program or a non-acting program. The adolescent acting group had greater baseline cognitive empathy than the adolescent non-actors, which improved after 1 year of training. The acting groups of both ages increased their scores on emotional empathy and on a measure of unspecified empathy post-training.

The studies reported above show that actors score higher than non-actors on empathy and suggest that acting training fosters growth in empathy, a key skill in healthy interpersonal relationships. When we tease apart the different kinds of empathy, however, the results are less clear. The current study uses a measure that allows for the differentiation of cognitive, emotional, and compassionate empathy in everyday life.

## Materials and Methods

### Participants

We recruited 88 participants, but excluded one non-acting student for not completing the quantitative measures. Included in the analyses were 87 participants^1^ (54 identified as women and 33 identified as men), ages 18–30 (*M* = 19.87, *SD* = 1.74).

Forty-three non-acting undergraduate students (hereafter called “non-actors”; 25 identified as women and 18 identified as men), ages 18–22 (*M* = 19.16, *SD* = 1.067), taking a psychology course were recruited from Boston College through an online participant recruitment tool and compensated one research participation credit.

Forty-four acting students (hereafter called “actors”; 37 from the Boston area, 5 from the Los Angeles American Academy of Dramatic Arts, and two from the University of Wisconsin-Milwaukee theater program; 29 identified as women and 15 identified as men), ages 18–30 (*M* = 20.57, *SD* = 1.99), were recruited through word of mouth (research assistants contacted acting teachers and their students) and compensated $20.

### Procedure

Participants first signed a consent form and then read a pre-selected monologue to themselves. They then completed a measure of dissociation. Next, they prepared the monologue and performed it in front of a camera. They were allowed to restart if they wanted (only one non-actor chose to). Immediately following the performance, they completed a measure of flow and the same measure of dissociation (in a counterbalanced order). Finally, they completed a measure of empathy and a demographic and acting questionnaire. Research assistants later rated the recordings.

Note that only dissociation was measured pre- and post-performance. We sought to replicate previous findings showing that actors have elevated levels of dissociation (Thomson et al., 2009; Thomson and Jaque, 2011, 2012b; Panero, 2015; Panero et al., 2020). Additionally, we wanted to test whether this dissociation would be affected by the acting experience. We did not measure flow pre-performance because there was no reason to believe that participants would be in a flow state upon arriving for the study. We also did not measure empathy pre-performance because we did not want to prime participants with the items on this measure.

### Materials

#### Monologue

All participants performed the same monologue chosen from *Bird of Prey* by Jim Grimsley (1999; see Supplementary Material for Recording Methods). Having all participants perform the same monologue removed the generalizability of results to other scripts; however, it provided experimental control over possible confounding variables. Though the character reciting this monologue is a teenage girl, when taken out of context, the monologue is appropriate for all ages, genders, and ethnicities. Furthermore, participants were asked to perform the monologue as their own gender, age, and ethnicity. The monologue was also chosen because the circumstances of the play appear ambiguous. This allows actors to use their imagination to fill in the gaps. It also prevents the raters from assuming that there is one correct way to perform this monologue. Participants were allotted 30 minutes to prepare the monologue and were allowed to look at the script during the performance so that memorization was not required. The participants’ performance time averaged 2 minutes and 44 seconds.

#### Dissociation

The Peritraumatic Dissociative Experiences Questionnaire (PDEQ; Marmar et al., 1998, 2004) assessed pre- and post-performance dissociation. The PDEQ was designed to be administered as soon as possible after a dissociation-inducing event for clear recall. This self-report questionnaire lists 10 dissociative experiences, such as “My sense of time changed – things seemed to be happening in slow motion” and “I felt as though I were a spectator watching what was happening to me, as if I were floating above the scene or observing it as an outsider.” Participants answered on a 5-point scale, where 1 = “not at all true” and 5 = “extremely true.” Total scores are calculated by averaging the individual item scores, and thus ranged from 1 to 5. Time 1 actors *α* = 0.73; non-actors *α* = 0.81. Time 2 actors *α* = 0.86; non-actors *α* = 0.91.

#### Flow

The Flow State Scale-2 (FSS-2; Jackson and Eklund, 2004) assessed flow solely post-performance. As with the PDEQ, the FSS-2 was designed to be administered as soon as possible after a flow-inducing activity for clear recall. This self-report questionnaire lists 36 flow experiences, such as “I was challenged, but I believed my skills would allow me to meet the challenge” and “I made the correct movements without thinking about trying to do so.” Participants answered on a 5-point scale, where 1 = “strongly disagree” and 5 = “strongly agree.” Total scores are calculated by averaging the individual item scores, and thus ranged from 1 to 5. Actors *α* = 0.85; non-actors *α* = 0.88.

The FSS-2 includes nine subscales based on Csikszentmihalyi’s (1990) conceptual dimensions of flow: autotelic experience (intrinsically rewarding experience), challenge-skill balance (personal skills meet the demands of the challenge), loss of self-consciousness, clear goals, transformation of time (the seeming of slowing or speeding up of time), sense of control, unambiguous feedback (knowledge that the activity is on track toward the goal), concentration on the task at hand (intense absorption), and action-awareness merging (feelings of being one with the activity). Each individual subscale score also ranged from 1 to 5.

#### Empathy

The Interpersonal Reactivity Index (IRI; Davis, 1980, 1983) assessed participants’ empathy. This is the only measure for which its authors report gender differences: women score higher than men. This self-report questionnaire lists 28 empathy experiences, such as “I try to look at everybody’s side of a disagreement before I make a decision” and “When I see someone being taken advantage of, I feel kind of protective toward them.” Participants answered on a 5-point scale, where 1 = “does not describe me well” and 5 = “describes me very well.” Total scores are calculated by averaging the individual item scores, and thus ranged from 1 to 5. Actors *α* = 0.60; non-actors *α* = 0.57.

The IRI includes four subscales: fantasy (tendency to imaginatively transpose the self into the feelings and actions of fictitious characters; i.e., emotional empathy), perspective taking (tendency to adopt the psychological point of views of others; i.e., cognitive empathy), empathic concern (feelings of sympathy and concern for others; i.e., compassionate empathy), and personal distress (feelings of personal anxiety in tense interpersonal situations; i.e., emotional empathy). Each individual subscale score also ranged from 1 to 5.

#### Demographic and Acting Questionnaire

The demographic and acting questionnaire contained 12 questions^2^ asking participants their age, gender identity, experience with the selected monologue, acting training and performance experience, and use of Method-like acting.

One actor reported having studied *Bird of Prey*, but provided no further information.

Responses to questions on acting training and on performance experience were categorized as “acting experience.” For acting training, participants received 1 point for taking one to two acting classes lasting less than one semester, 2 points for taking three or more acting classes lasting less than one semester or one to two acting classes lasting one semester or longer, or 3 points for taking more than two acting classes lasting one semester or longer. For performance experience, participants also received 1 point for participating in one to three productions as part of the ensemble, 2 points for participating in more than three productions as part of the ensemble or in one to two productions as a lead, or 3 points for participating in more than two productions as a lead. Therefore, scores for acting experience (the sum of acting training and performance experience) ranged from 0 to 6, with higher scores reflecting greater acting experience. Actors scored a mean of 3.88 and non-actors (with extracurricular acting experience) scored a mean of 0.60. Although we did not have a specific hypothesis about acting experience in particular, we included it (and the time taken to prepare the monologue for performance) as a control variable in Hypothesis 3 (described below).

Participants were categorized as Method actors if they endorsed at least one incidence of Method or Method-like acting, or boundary blurring between themselves and a character. Additionally, those who described finding their character by exploring emotions, biographical memories, or by other intangible means were categorized as Method actors. Thirty-eight actors were thus categorized as Method actors. Six actors were categorized as non-Method actors. Results did not change when these participants were excluded from analyses. Furthermore, since this cell was too small to perform any comparative statistical analyses, no hypotheses were developed for non-Method actors and all actors were included in analyses.

### Performance Ratings

Two undergraduate research assistants, blind to participant condition, independently rated the 87 performances in random order. One of the raters had 2 years of acting training; the other had no acting training (See Supplementary Material for Rater Training). The presence of six dimensions of acting was rated on a 5-point scale, where 1 = “do not agree at all” and 5 = “agree very strongly”: (1) This actor seemed to really “become” the character; (2) This actor seemed fully absorbed in acting this role; (3) This actor was believable as the character; (4) I would like to see a performance with this person as the lead actor; (5) I felt empathic toward the character this actor portrayed;^3^ (6) I would rate this actor as excellent, overall. These six items were designed to measure the participants’ ability to realistically portray the character through acting. The average of the six item scores was used as each rater’s score and the average of the two rater’s scores was used as each participant’s final rating score (See Supplementary Material for Validation of Rating Scores). To calculate inter-rater reliability, we computed an intraclass correlation coefficient, which showed that the raters’ scores had good reliability, ICC_(2,2)_ = 0.80, 95% CI [0.70, 0.87].

## Hypotheses

### Hypothesis 1

Actors should score higher than non-actors on dissociation (pre-performance), flow, and empathy (except for the personal distress subscale), as well, of course, on performance ratings.

We expected to replicate findings showing that actors score higher than non-actors on dissociation (Thomson et al., 2009; Thomson and Jaque, 2011, 2012b; Panero, 2015; Panero et al., 2020).

Non-actors are less likely than are actors to be able to engage with the monologue in a way that leads to flow because non-actors presumably have limited knowledge of what to do when asked to perform a monologue.

Actors plausibly use fantasy, perspective taking, and empathic concern (three of the four empathy subscales) when connecting with a character. The items on the personal distress subscale, however, describe feelings of anxiety in tense interpersonal situations. Scoring high on this subscale may indicate inferior emotion regulation, and some researchers believe that actors are adept in various emotion regulation skills (Ekman et al., 1983; Futterman et al., 1994; Pelletier et al., 2003; Gentzler et al., 2019). Moreover, the tendency to experience distress and discomfort in social situations is counterproductive to performing in front of an audience. Therefore, actors should score lower than non-actors on the personal distress subscale.

### Hypothesis 2

Dissociation scores of actors and non-actors should increase after the monologue performance. Similar to dissociation, acting requires that one behave differently from one’s real self and perhaps actors forget that they are merely acting. This may explain actors scoring higher than non-actors on dissociation when questioned retrospectively about their experiences while acting (Thomson et al., 2009; Thomson and Jaque, 2011, 2012b; Panero, 2015; Panero et al., 2020).

### Hypothesis 3

Performance ratings of actors should be predicted by dissociation (post-performance), flow, and empathy, over and above acting experience and the time taken to prepare the monologue for performance.

Because actors score higher than non-actors on dissociation (Thomson et al., 2009; Thomson and Jaque, 2011, 2012b; Panero, 2015; Panero et al., 2020), one could make the case that acting requires dissociation. If so, the performances of participants with high levels of post-performance dissociation should be highly rated.

Even if actors do not dissociate, they may still engage deeply with their acting, which may lead to or stem from flow. Therefore, the performances of participants with high levels of flow should be rated more positively.

It has been theorized that acting increases empathy because of how frequently actors embody and take on the points of view of many different characters (Musiker, 2015; Gross, 2018). It is also possible that highly empathic people gravitate toward acting. In either case, the performances of participants with high levels of empathy should be rated more positively than those with low levels.

## Results

Descriptives, means, and pairwise comparisons are reported in Table 1.

**Table 1:** Descriptives, mean scores (SDs), and pairwise comparisons between groups on all measures.

	Acting students (*n* = 44; *M*_age_ = 20.57; 29 women/15 men)	Non-acting students (*n* = 43; *M*_age_ = 19.16; 25 women/18 men)	Pairwise comparisons
Performance ratings	3.53 (1.041)	1.77 (0.89)	*p* < 0.001, *d* = 1.82
Dissociation pre-performance	1.92 (0.56)	1.82 (0.61)	*p* = 0.13, *d* = 0.17
Post-performance	2.08 (0.76)	1.98 (0.86)	*p* = 0.57, *d* = 0.12
Flow overall score	3.63 (0.38)	3.31 (0.42)	*p* = 0.002, *d* = 0.80
Autotelic experience	3.97 (0.63)	3.23 (0.86)	*p* < 0.001, *d* = 0.98
Challenge-skill balance	3.78 (0.65)	3.20 (0.66)	*p* < 0.001, *d* = 0.89
Loss of self-consciousness	3.61 (0.98)	2.92 (0.98)	*p* = 0.001, *d* = 0.70
Clear goals	3.54 (0.74)	3.19 (0.77)	*p* = 0.032, *d* = 0.46
Transformation of time	3.54 (0.65)	3.34 (0.75)	*p* = 0.20, *d* = 0.28
Sense of control	3.54 (0.69)	3.35 (0.72)	*p* = 0.21, *d* = 0.27
Unambiguous feedback	3.39 (0.85)	3.32 (0.72)	*p* = 0.67, *d* = 0.089
Concentration on task at hand	3.70 (0.93)	3.67 (0.73)	*p* = 0.89, *d* = 0.036
Action-awareness merging	3.59 (0.66)	3.57 (0.68)	*p* = 0.92, *d* = 0.029
Empathy overall score	3.53 (0.41)	3.13 (0.48)	*p* < 0.001, *d* = 0.90
Fantasy	4.094 (0.61)	3.29 (0.85)	*p* < 0.001, *d* = 1.087
Perspective taking	3.64 (0.78)	3.25 (0.64)	*p* = 0.013, *d* = 0.55
Empathic concern	3.85 (0.67)	3.52 (0.70)	*p* = 0.026, *d* = 0.48
Personal distress	2.56 (0.62)	2.48 (0.69)	*p* = 0.57, *d* = 0.12
Acting experience	3.89 (1.66)	0.60 (1.20)	*p* < 0.001, *d* = 2.27
Seconds taken to prepare monologue for performance	841.01 (488.090)	533.27 (377.058)	*p* = 0.002, *d* = 0.71

### Results for Hypothesis 1

To assess whether actors score higher than non-actors on performance ratings, dissociation (pre-performance), flow, and empathy (except for the personal distress subscale), the following three analyses were performed.

#### Analysis 1

**Figure 1:**
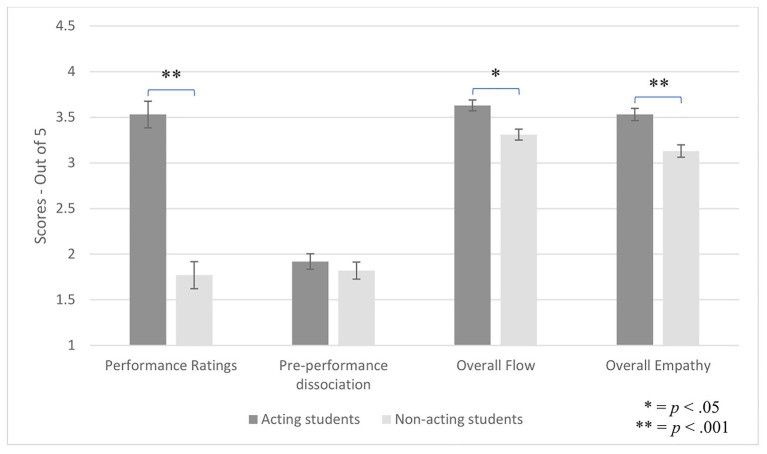
Group differences on performance ratings, dissociation (pre-performance), flow, and empathy.

A multivariate analysis of covariance (MANCOVA), controlling for gender and age, showed a main effect of group on performance ratings, dissociation (pre-performance), flow, and empathy, *F*(4, 80) = 14.18, *p* < 0.001, Wilks’ *Λ* = 0.59, *η*_p_^2^ = 0.42. As hypothesized, actors scored higher than non-actors on performance ratings, *F*(1, 83) = 45.59, *p* < 0.001, *d* = 1.82, flow, *F*(1, 83) = 9.86, *p* = 0.002, *d* = 0.80, and empathy, *F*(1, 83) = 14.35, *p* < 0.001, *d* = 0.90. Contrary to hypothesis, no group differences emerged for pre-performance dissociation, *F*(1, 83) = 2.39, *p* = 0.13 (see Figure 1).

#### Analysis 2

**Figure 2:**
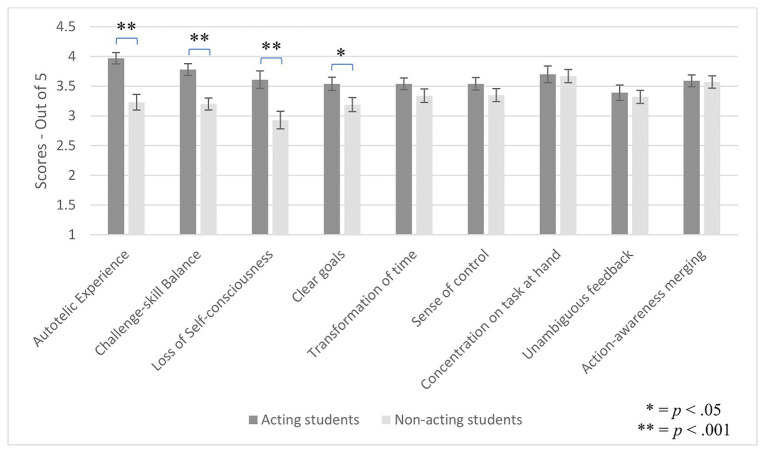
Group differences on flow subscales.

The effect of group on flow subscales was analyzed with a repeated measures ANOVA. There was a main effect of flow subscale, *F*(8, 680) = 3.18, *p* = 0.002, *η*_p_^2^ = 0.036, and a main effect of group, *F*(1, 85) = 14.16, *p* < 0.001, *η*_p_^2^ = 0.14, showing that the means differed across flow subscales and across groups. Group interacted with flow subscale, *F*(8, 680) = 3.71, *p* < 0.001, *η*_p_^2^ = 0.042. To explore this interaction, independent samples *t*-tests were performed. Actors scored higher than non-actors on four flow subscales: autotelic experience, *t*(85) = 4.60, *p* < 0.001, *d* = 0.98, challenge-skill balance, *t*(85) = 4.16, *p* < 0.001, *d* = 0.89, loss of self-consciousness, *t*(85) = 3.29, *p* = 0.001, *d* = 0.70, and clear goals, *t*(85) = 2.18, *p* = 0.032, *d* = 0.46. There were no group differences on the remaining five flow subscales: transformation of time, *t*(85) = 1.31, *p* = 0.20, sense of control, *t*(85) = 1.27, *p* = 0.21, concentration on the task at hand, *t*(85) = 0.14, *p* = 0.89, unambiguous feedback, *t*(85) = 0.43, *p* = 0.67, and action-awareness merging, *t*(85) = 0.11, *p* = 0.92. Thus, actors scoring higher on four of the nine flow subscales drove the interaction of group by flow subscale (see Figure 2).

#### Analysis 3

The effect of group and gender on empathy subscales was then analyzed. Gender was included because previous research established that women score higher than men on all empathy subscales (all *p*s < 0.001; Davis, 1980), and there were 7.41% more women in the actor group than in the non-actor group. A 2 (group) by 2 (gender) by 4 (empathy subscales) ANOVA with repeated measures on the empathy subscale factor yielded a main effect of empathy subscale, *F*(3, 249) = 66.60, *p* < 0.001, *η*_p_^2^ = 0.45, showing that means differed across empathy subscales.

**Figure 3:**
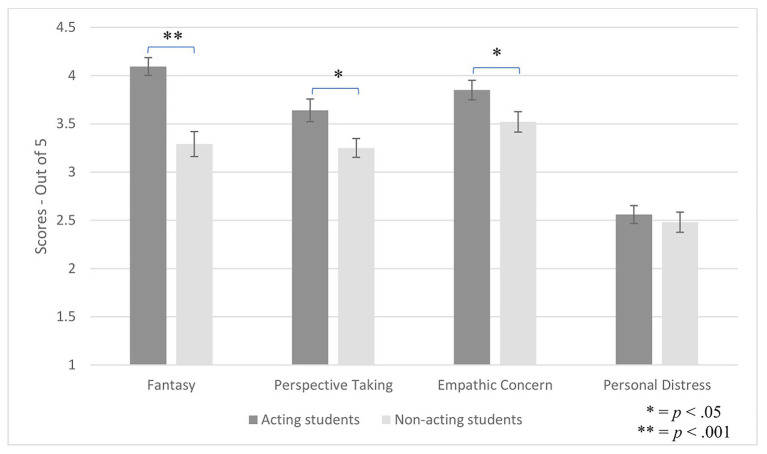
Group differences on empathy subscales.

Group interacted with empathy subscale, *F*(3, 249) = 3.39, *p* = 0.019, *η*_p_^2^ = 0.039. To explore this interaction, we performed independent samples *t*-tests. As hypothesized, actors scored higher than non-actors on fantasy, *t*(85) = 5.092, *p* < 0.001, *d* = 1.087, perspective taking, *t*(85) = 2.53, *p* = 0.013, *d* = 0.55, and empathic concern, *t*(85) = 2.27, *p* = 0.026, *d* = 0.48. No group differences emerged on personal distress, *t*(85) = 0.57, *p* = 0.57 (see Figure 3).

**Figure 4:**
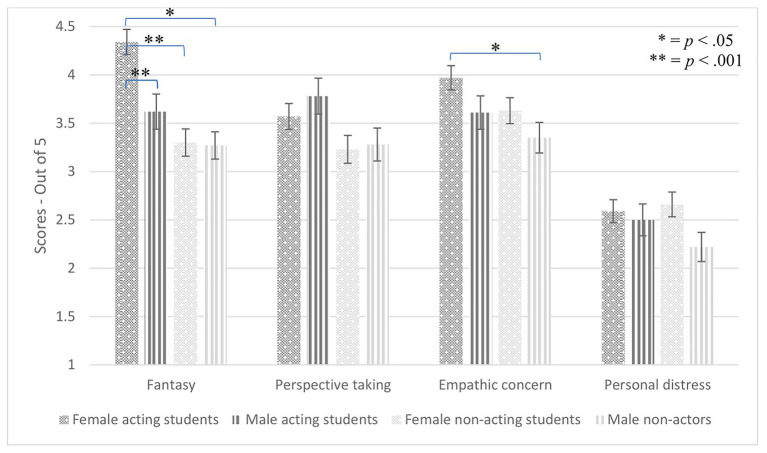
Subgroup differences on empathy subscales.

Gender also interacted with the empathy subscale, *F*(3, 249) = 2.92, *p* = 0.035, *η*_p_^2^ = 0.034, and there was a three-way interaction of empathy subscale, group, and gender, *F*(3, 249) = 2.81, *p* = 0.040, *η*_p_^2^ = 0.033. To explore this interaction, we performed four ANOVAs by subgroup (four levels, female actors, male actors, female non-actors, and male non-actors). The fantasy subscale yielded a significant effect of subgroup, *F*(3, 83) = 12.96, *p* < 0.001, *η*_p_^2^ = 0.32. LSD *post-hoc* pairwise comparisons revealed that female actors scored higher than male actors (*p* = 0.002, *d* = 1.40), female non-actors (*p* < 0.001, *d* = 1.46), and male non-actors (*p* < 0.001, *d* = 1.58). Additionally, empathic concern yielded a significant effect of subgroup, *F*(3, 83) = 3.36, *p* = 0.023, *η*_p_^2^ = 0.11. LSD post-hoc pairwise comparisons revealed that female actors scored higher than male non-actors (*p* = 0.003, *d* = 0.84). Neither of the other ANOVAs revealed a significant effect of subgroup: perspective taking, *F*(3, 83) = 2.40, *p* = 0.073, *η*_p_^2^ = 0.080; personal distress, *F*(3, 83) = 1.85, *p* = 0.15, *η*_p_^2^ = 0.063. Thus, female actors scoring high on fantasy and empathic concern carried the effect of the three-way interaction of empathy subscale, group, and gender (see Figure 4).

No gender differences were found on any of the other measures.

### Results for Hypothesis 2

**Figure 5:**
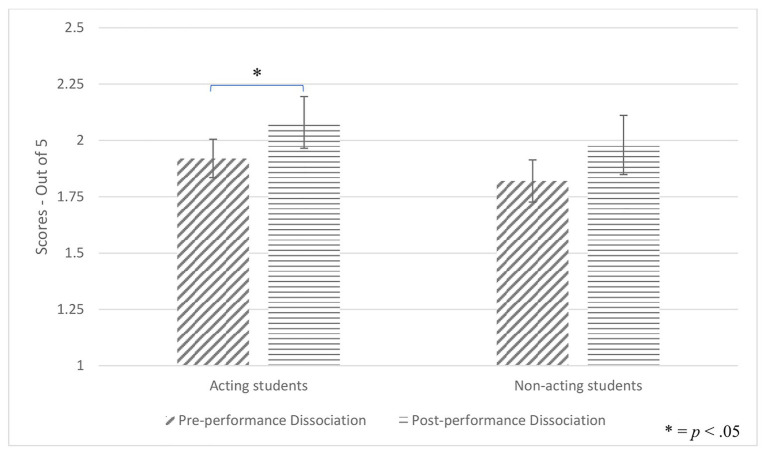
Pre- and post-performance dissociation per group.

Paired samples *t*-tests examined whether dissociation of both groups increased immediately after performing. Dissociation increased significantly in actors, *t*(43) = 2.09, *p* = 0.042, *d* = 0.25, but only marginally significantly in non-actors, *t*(42) = 1.77, *p* = 0.083 (see Figure 5).

### Results for Hypothesis 3

To examine whether performance ratings are predicted by dissociation (post-performance), flow, and empathy, over and above acting experience and time taken to prepare the monologue for performance, linear multiple regression analyses were performed for all participants and one for each group separately.

The regression using all participants was significant, *F*(5,78) = 32.67, *p* < 0.001, *R*^2^ = 0.68. Surprisingly, acting experience was the only significant predictor (*β* = 0.68, *t* = 9.45, *p* < 0.001), explaining 36.97% of the variance over and above the other predictors.

Since acting experience and group were strongly correlated (*r* = 0.75, *p* < 0.001), we conducted the same analysis for each group separately. For actors, the regression was significant, *F*(5,37) = 6.78, *p* < 0.001, *R*^2^ = 0.48. Again, acting experience was the only significant predictor (*β* = 0.68, *t* = 5.66, *p* < 0.001), explaining 45.29% of the variance over and above the other predictors. For non-actors, the regression was also significant, *F*(5,35) = 4.19, *p* = 0.004, *R*^2^ = 0.38. Once more, acting experience was the only significant predictor (*β* = 0.38, *t* = 2.71, *p* = 0.010), explaining 13.10% of the variance over and above the other predictors. Contrary to hypothesis, these results show that neither the participants’ self-reported experiences of dissociation, flow, and empathy, nor the time they took to prepare the monologue for performance, independently predicted the performance ratings received.

#### Exploratory Post-hoc Analysis for Hypothesis 3

Since performance ratings and acting experience were strongly correlated (*r* = 0.79, *p* < 0.001), we removed acting experience as a predictor and ran a linear multiple regression analysis regressing dissociation (post-performance), flow, empathy, and the time taken to prepare the monologue for performance on performance ratings. The regression was not significant, *F*(4,38) = 0.25, *p* = 0.91, *R*^2^ = 0.025.

## Discussion

We examined dissociation (a potentially negative experience), flow (a positive experience), and empathy (an important social skill) in acting students, three characteristics that may enable them to achieve the goal set by Method acting teacher Strasberg (1987) to create real emotion experiences on stage. We also examined the extent to which these characteristics contributed to quality acting, as rated by independent observers. Our hope is that these results may be useful not only for research psychologists but also for the acting community.

### How Do Acting and Non-acting Students Differ in Dissociation, Flow, and Empathy?

Actors scored higher than non-actors on flow (and some flow subscales) and empathy (and some empathy subscales), as predicted, but not on pre-performance dissociation. However, actors’ dissociation increased immediately post-performance.

#### Dissociation

No group differences emerged on pre-performance dissociation. This result is inconsistent with previous research in which actors scored significantly higher than non-actors on dissociation (Thomson et al., 2009; Thomson and Jaque, 2011, 2012b; Panero, 2015; Panero et al., 2020). The discrepancy may be due to the different measures used. The current study used the PDEQ, which was designed to be administered after a dissociation-inducing event, to probe for dissociation pre- and post-performance. Therefore, this questionnaire might not have been able to capture any existing baseline dissociation. Although this is a limitation in our study, it allowed us to quantify changes in dissociation due to our acting manipulation.

Previous studies asked actors to complete the Dissociative Experiences Scale-II (DES-II; Carlson and Putnam, 1993), a self-report measure used by clinicians as a screening tool, to reflect on previous dissociative experiences. Retrospective thinking may lead to erred responding. Furthermore, to an actor, the items on the DES-II could seem to describe experiences that are encouraged in acting classes, such as becoming so involved in a fantasy that it feels real. Perhaps actors have a response bias toward questions that they think indicate good acting. Alternatively, perhaps our chosen monologue and/or audition-like manipulation did not induce the extreme emotional acting experiences that actors recalled during other studies. Thus, no direct comparisons can be made between the current study and previous ones. Future research could investigate how to best capture and compare the dissociative experiences of actors, both while acting and during daily activities.

Although actors and non-actors had similar levels of pre-performance dissociation, an interesting and potentially revealing finding did emerge. The fact that actors reported higher levels of dissociation immediately post-performance compared to baseline, suggests that actors do dissociate more than non-actors as they step into the shoes of a character. Nevertheless, since this increase yielded a small effect size (*d* = 0.25), no definitive interpretation can be drawn.

The fact that non-actors’ dissociation only marginally increased can be explained in three ways: (1) not everyone who attempts to act immediately dissociates because dissociation is learned with acting training; (2) acting leads to dissociation only in those with a predisposition toward dissociation (e.g., actors); or (3) non-actors were not engaged enough with the acting to trigger a dissociative experience. Future research paralleling the work done with visual art education (Hetland et al., 2007) and music education (Hogan and Winner, in press) is currently underway to investigate the habits of mind taught in acting classes and determine whether acting training promotes dissociation. Preliminary analyses do not show that acting teachers explicitly teach concepts related to dissociation (Goldstein and Young, 2019). However, should final results reveal that acting teachers explicitly or implicitly teach students to dissociate, then perhaps acting training should be re-evaluated to ensure this dissociation does not affect students’ psychological well-being.

#### Flow

Results showed that actors reported higher levels of specific components of flow after their performance than did non-actors. Consistent with Allen (2001) and Martin and Cutler (2002), actors’ flow was comprised of autotelic experience (intrinsically rewarding experience), challenge-skill balance (personal skills meet the demands of the challenge), loss of self-consciousness, and clear goals. These findings suggest that the *Bird of Prey* monologue was at the right level of challenge (not too easy or too difficult).

Also consistent with Martin and Cutler (2002), transformation of time (the seeming of slowing or speeding up of time) and unambiguous feedback (knowledge that the activity is on track toward the goal) were not particularly important for the actors’ flow to occur. These findings, however, are inconsistent with Allen (2001) and Panero et al. (2020) who did find evidence of these components of flow in actors. These inconsistencies may be due to differences in the samples. Allen (2001) studied professional actors, while Panero (2015) and Panero et al. (2020) studied acting students in a conservatory training program. In contrast, the current study examined non-conservatory acting students – hence possibly less trained actors.

Nevertheless, all of the studies do converge in showing that actors do not experience flow in terms of sense of control, concentration on the task at hand (intense absorption), or action-awareness merging (feelings of being one with the activity). Future research could investigate whether teaching acting techniques to non-actors would help them to have more peak positive psychological experiences during specific activities.

#### Empathy

The finding that actors reported higher levels of empathy in their everyday lives than non-actors is consistent with previous studies (Chandler, 1973; Nettle, 2006; Goldstein et al., 2009–2010; Goldstein and Winner, 2012; Dumas et al., 2020). The subscale results revealed that the actors’ empathy was comprised of all three types of empathy: emotional empathy (as measured by the fantasy subscale – tendency to imaginatively transpose the self into the feelings and actions of fictitious characters), cognitive empathy (as measured by the perspective taking subscale – tendency to adopt the psychological point of views of others), and compassionate empathy (as measured by the empathic concern subscale – feelings of sympathy and concern for others). Perhaps acting demands that actors make use of fantasy, perspective taking, and empathic concern to connect with a character. Future research could develop protocols for non-actors (e.g., drama therapy patients) to use acting techniques to improve their empathic abilities and enhance their social lives.

Female actors scored higher than all other participants on fantasy and higher than male non-actors on empathic concern. Because Davis (1980) established that women score higher than men on all of the empathy subscales, it is surprising that female actors did not score higher than other participants on perspective taking. Not surprising, however, is that actors did not score high on personal distress (feelings of anxiety in tense interpersonal situations). This finding provides evidence for the assumption of some researchers that actors are adept in various emotion regulation skills (Ekman et al., 1983; Futterman et al., 1994; Pelletier et al., 2003; Gentzler et al., 2019). It is important to note, however, that the poor reliability across items in our samples might have reduced the measure’s ability to accurately reflect the construct of empathy.

### What Predicts Performance Ratings?

As expected, actors scored higher than non-actors on performance ratings. But what factors predict variation in performance ratings of actors? When acting experience was included as a predictor along with dissociation (post-performance), flow, empathy, and the time taken to prepare the monologue for performance, only acting experience significantly predicted performance ratings for all participants, as well as for each group separately. Of course, actors had more acting experience than did non-actors. When acting experience was excluded as a predictor, all of the predictors again failed to predict quality acting. These results fit with the old adage “practice makes perfect.”

The ability to merge with a character (as measured by dissociation, flow, and empathy) did not lead to higher quality acting. This is interesting because Method acting, arguably the most popular type of acting taught in the United States, calls for actors to “become” their characters (i.e., to dissociate) and to “be in the moment” (i.e., be in flow) by living and feeling (i.e., being empathic) as their characters (Konijn, 1997; Krasner, 2000; Knowles, 2004; Lutterbie, 2011; Kemp, 2012; Ohikuare, 2014). Both anecdotal (Konijn, 1997; Lyall, 2007; Freyer, 2012; Buitenhuis and Murray, 2017) and scientific (Thomson et al., 2009; Thomson and Jaque, 2011, 2012b; Panero, 2015; Panero et al., 2020) evidence suggests that merging with a character may be dangerous and lead to pathology. Although, of course, true experimental studies are needed to draw definitive conclusions on causality, the current findings suggest that Method acting might not be necessary to give a convincing performance.

Although we were glad not to find a relationship between the negative experience of pathological dissociation and performance ratings, we were surprised that neither flow (considered a measure for optimal performance) or empathy were significant predictors. As with all self-report measures, response bias may be a factor in these results. It is possible that student actors like to think of themselves as empathic people who enter flow states while acting rather than actually being an especially empathic group with the ability to experience flow. A limitation of the present research was a lack of a professional actor comparison group. Dumas et al. (2020) showed that professional actors (i.e., experienced actors) score higher than student actors (i.e., less experienced actors) on a number of characteristics (e.g., compassion, grit, and divergent thinking). Future research may explore whether dissociation, flow, and empathy, as well as other aspects of acting experience not addressed in this study (e.g., feeling comfortable in front of a camera), predict performance outcomes across actors with varying experience.

Additionally, future research could compare whether other approaches to acting that are less emotionally taxing (and possibly psychologically healthier) result in a differing quality of acting. One of these techniques is the “outside-in” approach, popular with British actors, which encourages less focus on emotions and more on the exterior, physical qualities of the characters (Konijn, 1997; Benedetti, 2007). American actors may want to embrace the words of British actor, Laurence Oliver, who said to Dustin Hoffman, an American actor, when he allegedly stayed up for three nights to feel like his character in *Marathon Man*: “Dear boy, you look absolutely awful. Why don’t you try *acting*? It’s so much easier” (Clines, 1987, p. C13).

### Conclusion

This research shines a spotlight on an interesting population – actors. Actors constitute a population whose product we are eager to consume, yet we do not know much about how they do what they do. The results reveal something about the personality traits of actors and something about how actors are affected by acting. Although results from small sample sizes should be subjected to replication, the large effect sizes achieved throughout this study indicate robust findings. More research on acting is clearly called for. After all, acting is universal across human cultures. Any theory of human nature needs to be able to account for why humans act, what it takes to do it, and how acting affects the actor. As Wilson (1985), a research psychologist and opera singer, stated:

If psychology is “the science of behavior and experience” and theater is “a mirror to life,” each should have something to offer the other… actors, singers, musicians, directors, even the theater-going public, can benefit from a survey of what the life sciences have to say about performance, while psychologists can equally profit from investigating what theater tells about human nature (Preface, para. 1).

## Data Availability Statement

The raw data supporting the conclusions of this article will be made available by the authors, without undue reservation.

## Ethics Statement

The studies involving human participants were reviewed and approved by Boston College. The participants provided their written informed consent to participate in this study.

## Author Contributions

MEP: concept of study, data collection, data analyses, and interpretation. MEP and EW: design of study and manuscript writing. Both the authors contributed to the article and approved the submitted version.

## Acknowledgments

We would like to thank Hiram Brownell, Andrea Heberlein, and Andrew Sofer of Boston College for their guidance on this study.

### Conflict of Interest

The authors declare that the research was conducted in the absence of any commercial or financial relationships that could be construed as a potential conflict of interest.

## Footnotes

^1^Sample sizes were based on Panero (2015) in which dissociation between student actors and non-acting students was compared, yielding an effect size of Cohen’s *d* = 0.70. An analysis on G*Power 3.1.9.2 determined that in order to obtain a power of *d* = 0.8 with *α* error probability of 0.05 the minimum sample size per group should be 35.

^2^Note that the responses to question 11 were not analyzed for this study. This question asked about differences between the emotions experienced while acting during rehearsal, during performance, during acting class, and during an audition. We concluded that this question was not clearly phrased because the responses were uninformative and did not fit into a manageable scoring scheme.

^3^Dimension number 5 can only be used to rate actors playing a character for which the audience is supposed to feel empathy; we selected our monologue with this in mind.
