# Supplementary material for: Rating the Acting Moment: Exploring Characteristics for Realistic Portrayals of Characters
Source: Front Psychol. 2021 Feb 4;11:615311. doi: 10.3389/fpsyg.2020.615311 (PMC7889958; doi:10.3389/fpsyg.2020.615311)
Supplement: Supplementary file 1 [file Data_Sheet_1.pdf]

Supplemental Materials

Recording Methods

All participants were recorded in comparable rooms and all recordings were made by research assistants on smart phone cameras (i.e., LG Nexus 5, iPhone 6, iPhone 6S, iPhone 6SE, iPhone 7), which had comparable technology (1080p-2160p resolution and 8-16 megapixels), and which were stabilized on a tripod. Research assistants were asked to avoid shadows, but no additional lights or microphone were provided. As in ideal self-recorded auditions (Hacker, 2011; Ross, 2014), the camera lens was set at the participant's eye level, with the participant in a medium frame, and with a blank background. Participants were instructed to direct the monologue straight ahead, into the camera. Certain videos were edited to adjust the frame, cut out dialogue between the research assistant and participant, cut out participants' identifying information, and/or cut out a participant's first attempt at the monologue. All recordings were saved in a protected file and deleted from the phones.

Rater Training

Prior to rating the participants, the two independent raters were simultaneously trained by watching seven video clips of professional performances: three Academy Award winning actors (Jack Lemmon, Jeff Bridges, and Ellen Burstyn) and four Golden Raspberry Award winning actors (Steven Seagal, Burt Reynolds, Judd Nelson, and Tanya Roberts). The Golden Raspberry Awards (also known as the Razzies or Razzie Awards) are mock awards in recognition of the worst in film, which have been recognized by numerous international journalists and film industry professionals (Lindrea, 2007; Marrs, 2009). As with the participant videos, the training videos showed the main actor in a medium frame. These award-winning actors were chosen as examples of excellent and of poor acting.

The specific training video clips were also chosen because they were old enough to likely be unrecognized by the raters, but not so old as to seem dated. Nevertheless, the raters mentioned that the video quality, editing style, background music, and acting style appeared antiquated. This unexpected response served as an additional training opportunity to focus the raters' attention solely on the six acting dimensions relevant to this study. Additionally, raters were instructed to not evaluate the study participants on appearance or skill at memorization.

Both raters watched all seven training video clips and independently rated the performances as they would later do for the participant videos. Unlike during the rating of the participants, however, the raters were provided with background information on the plot of the movie and, after rating each video, there was an open discussion about how each rater rated each video and why. To validate this training protocol, an ANOVA with type of award as the independent variable was conducted on the scores of the raters. The results showed that the raters rated the Oscar winners ($M = 4.67$, $SD = .29$) significantly higher than the Razzie winners ($M = 2.92$, $SD = .79$), $F(1,5) = 12.911$, $p = .016$, $d = 2.94$, demonstrating that the raters agreed with the judgments of the professional acting community.

Finally, after completing the original seven training videos, two additional videos for training were acquired. One participant video was re-purposed as a training video because the participant did not complete the quantitative measures and was thus excluded from all analyses (as mentioned in the Participants section). This video also served as a demonstration of what the raters would see after training. Furthermore, since the training video with Judd Nelson also starred Razzie Award winner for Worst Actress, Ally Sheedy, it was re-used as a training video with a focus on her. Both raters watched both video clips, independently rated them on the six dimensions of acting, and then had an open discussion about each one.

To calculate inter-rater reliability for the nine training videos, we used the same method as with the participant videos and computed an intraclass correlation coefficient which showed that the raters' scores had good reliability, $ICC_{(2,2)} = .88$, 95% CI [.46, .97].

Once rating of the participant videos began, inter-rater reliability was repeatedly calculated following the completion of small sets of video ratings. This technique was adopted as ongoing training to ensure that the raters maintained their reliability throughout the multiple rating sessions. Rating discrepancies were solved through discussion; however, no changes were made to the numeric values of the initial ratings. Table S1 shows the intraclass correlation coefficients for each set of video ratings, after averaging across the six item scores so that each video received one score from each rater.

Table S1

Intraclass Correlation Coefficients for Sets of Participant Videos

Set of video numbers	$ICC_{(2,2)}$	95% CI
1-29	.85	.68, .93
30-39	.89	.57, .97
40-49	.96	.84, .99
50-59	.99	.98, .99
60-69	.85	.38, .96
70-79	.89	.54, .97
80-87	.82	.12, .97

Validation of Rating Scores

To examine the appropriateness of collapsing across the six item scores to achieve one score per rater, Cronbach's alpha and a principal component analysis with no rotation was implemented. Since each rater's six scores was used for each participant, each participant appeared as two different cases. Cronbach's alpha showed excellent internal consistency at .98. For the principal component analysis, all six questions had loadings of .96 or higher on the first principal component, which accounted for 93.88% of the variance. The Eigenvalues for the remaining components were less than .15. The same results emerged when the analyses for each rater was performed separately.

References

Hacker, J. (2011). *Audition on camera: An actor's guide*. Routledge.

Lindrea, V. (2007, February 24). *Blowing raspberries at Tinseltown*. BBC News. http://news.bbc.co.uk/2/hi/entertainment/6392701.stm

Marrs, J. (2009, February 25). *They have no excuse to be as bad as they are*. The Guardian. https://www.theguardian.com/film/2009/feb/24/razzies-interview-oscars

Liem, C. (2013, June 17). 8 tips to make your self-taped audition look its best. *Backstage*. https://www.backstage.com/magazine/article/tips-self-taping-auditions-15753/
